# 15-Deoxy-Δ^12,14^-prostaglandin J_2_ Upregulates VEGF Expression via NRF2 and Heme Oxygenase-1 in Human Breast Cancer Cells

**DOI:** 10.3390/cells10030526

**Published:** 2021-03-02

**Authors:** Eun-Hee Kim, Su-Jung Kim, Hye-Kyung Na, Wonshik Han, Nam-Jung Kim, Young-Ger Suh, Young-Joon Surh

**Affiliations:** 1 College of Pharmacy and Institute of Pharmaceutical Sciences, CHA University, Seongnam 13488, Korea; ygsuh@snu.ac.kr; 2 Tumor Microenvironment Global Core Research Center, College of Pharmacy, Seoul National University, Seoul 08826, Korea; nynna79@snu.ac.kr; 3 Department of Food Science and Biotechnology, College of Knowledge-Based Services Engineering, Sungshin Women’s University, Seoul 01133, Korea; nhk1228@sungshin.ac.kr; 4 Cancer Research Institute, Seoul National University, Seoul 03080, Korea; hanw@snu.ac.kr; 5 Department of Surgery, Seoul National University College of Medicine, Seoul 03080, Korea; 6 College of Pharmacy, Kyung Hee University, Seoul 02447, Korea; kimnj@khu.ac.kr; 7 Department of Molecular Medicine and Biopharmaceutical Sciences, Graduate School of Convergence Science and Technology, Seoul National University, Seoul 08826, Korea

**Keywords:** 15-Deoxy-Δ^12,14^-prostaglandin J_2_, NRF2, heme oxygenase-1, VEGF, breast cancer, MCF-7 cells, angiogenesis

## Abstract

There is a plethora of evidence to support that inflammation is causally linked to carcinogenesis. Cyclooxygenase-2 (COX-2), a rate-limiting enzyme in the biosynthesis of prostaglandins, is inappropriately overexpressed in various cancers and hence recognized as one of the hallmarks of chronic inflammation-associated malignancies. However, the mechanistic role of COX-2 as a link between inflammation and cancer remains largely undefined. In this study, we found that 15-deoxy-Δ^12,14^-prostaglandin J_2_ (15d-PGJ_2_), one of the final products of COX-2, induced upregulation of vascular endothelial growth factor (VEGF) and capillary formation and migration through nuclear factor erythroid 2-related factor 2 (NRF2)-dependent heme oxygenase-1 (HO-1) induction in MCF-7 cells. Analysis of the publicly available TCGA data set showed that high mRNA levels of both COX-2 and NRF2 correlated with the poor clinical outcomes in breast cancer patients. Moreover, human tissue analysis showed that the levels of 15d-PGJ_2_ as well the expression of COX-2, NRF2, and HO-1 were found to be increased in human breast cancer tissues. In conclusion, the elevated levels of 15d-PGJ_2_ during inflammatory response activate VEGF expression through NRF2-driven induction of HO-1 in human breast cancer cells, proposing a novel mechanism underlying the oncogenic function of 15d-PGJ_2_.

## 1. Introduction

Heme oxygenase-1 (HO-1) catalyzes the conversion of heme to carbon monoxide (CO) and bilirubin with a concurrent release of iron. HO-1 expression is relatively low under basal conditions but elevated by stress-inducing agents such as UV radiation, heavy metals, lipopolysaccharides, and reactive oxygen/nitrogen species (ROS/RNS) [1,2]. The physiological functions of HO-1 are mainly associated with a fundamental adaptive and defensive response against oxidative stress, inflammation, and other injuries [3]. Inhibitors of HO-1 including zinc protoporphyrin IX (ZnPP) and tin protoporphyrin often exacerbate the pathogenesis of some disorders, such as graft rejection [4] and ischemia-reperfusion injury [5], in which systemic inflammation operates. Conversely, pharmacological HO-1 inducers and selective overexpression of HO-1 by genetic manipulation confer anti-inflammatory and other cytoprotective effects in cultured cells and in a variety of animal models of various diseases [6].

However, HO-1 also participates in the pathogenesis and progression of several types of malignancies. HO-1 is extensively expressed in various precancerous conditions and tumor cells including melanoma [7], renal adenocarcinoma [8], lymphosarcoma [9], benign prostatic hyperplasia and prostate cancer [10], and acute hepatitis and hepatoma [11]. In addition, administration of the HO-1 inhibitor ZnPP significantly suppressed the growth of tumors [12]. Upregulation of HO-1 has been shown to contribute to the angiogenesis in pancreatic carcinoma [13] and resistance to apoptotic stimuli in gastric cancer cells [14]. Furthermore, HO-1 overexpression increased viability, proliferation, and angiogenic potential of melanoma cells and augmented metastasis of tumor-bearing mice [15]. Induction of HO-1 is hence likely to be associated with carcinogenesis under certain conditions [16].

15-Deoxy-Δ^12,14^-prostaglandin J_2_ (15d-PGJ_2_), formed by cyclooxygenase-2 (COX-2) in inflamed site, has pro-resolving and anti-inflammatory activity [17]. The anti-inflammatory effects of 15d-PGJ_2_ have been attributed to its interruption of nuclear factor-κB (NF-κB) and subsequent blockade of proinflammatory gene expression [18,19]. Although the majority of the published studies imply the inhibitory effects of 15d-PGJ_2_ on carcinogenesis, there are some reports highlighting the opposite effect of 15d-PGJ_2_ on the development and progression of tumors. Thus, 15d-PGJ_2_ significantly enhanced the rate of formation, the size, and the vascularization of papillomas in a murine carcinogenesis model [20]. In another study, 15d-PGJ_2_ and its precursor PGJ_2_ induced the proliferation of COX-2-depleted colorectal cancer (HCA-7) cells at a nanomolar concentration [21]. However, the precise mechanisms responsible for tumor proliferative effects of 15d-PGJ_2_ remain incompletely clarified.

Vascular endothelial growth factor (VEGF) is well known as a master regulator of angiogenic switch [22]. Interestingly, VEGF upregulates HO-1 in vascular endothelial cells, while HO-1 may also regulate the synthesis and activity of VEGF, thus constituting a positive feedback loop [22]. In addition, 15d-PGJ_2_ was found to stimulate the expression of VEGF in endothelial cells, human androgen-independent PC3 prostate cancer cells, and the 5637 urinary bladder carcinoma cell line [23,24]. The upregulation of VEGF by 15d-PGJ_2_ was accompanied by activation of peroxisome proliferator-activated receptor gamma (PPARγ) [25]. However, the VEGF promoter does not seem to harbor the consensus sequence of the PPAR responsive element [26,27]. Interestingly, VEGF upregulation by 15d-PGJ_2_ could be mimicked by the induction of HO-1 expression in macrophages [25]. 15d-PGJ_2_ was previously reported to induce the expression of HO-1 in MCF-7 human breast cancer cells [28].

The nuclear factor erythroid 2-related factor 2 (NRF2) is a transcription factor responsible for maintenance of cellular redox balance by inducing antioxidant and other cytoprotective gene expression [29]. *HO-1* is a prototypic NRF2 target gene, and the aberrant hyperactivation of signaling mediated by NRF2/HO-1 axis contributes to tumor progression, aggressiveness, chemoresistance, and poor prognosis [30]. Herein, we report that 15d-PGJ_2_ induces VEGF expression and angiogenesis in human breast cancer cells through Nrf2-mediated upregulation of HO-1.

## 2. Materials and Methods

### 2.1. Chemical and Biochemical Reagents

15d-PGJ_2_, 9,10-dihydro-15d-PGJ_2_, and biotinylated 15d-PGJ_2_ were purchased from Cayman Chemical Co. (Ann Arbor, MI, USA). Biotinylated 9, 10-dihydro-15d-PGJ_2_ was kindly provided from Dr. Young-Ger Suh (Cha University, Seongnam, South Korea). RPMI 1640 medium and fetal bovine serum (FBS) were obtained from Gibco BRL (Grand Island, NY, USA). Antibodies against NRF2, HO-1, VEGF, and actin were obtained from Santa Cruz Biotechnology, Inc. (Santa Cruz, CA, USA). ZnPP was provided from OXIS International Inc. (Portland, OR, USA). The anti-rabbit and anti-mouse horseradish peroxidase conjugated secondary antibodies were purchased from Zymed Laboratories (San Francisco, CA, USA). The enhanced chemiluminescence (ECL) and [γ-^32^P] ATP were supplied from Amersham Pharmacia Biotech (Buckinghamxhire, UK). Antioxidant responsive element (ARE) or GC-mutated ARE-driven luciferase porter plasmid and dominant-negative (DN) NRF2 were kindly provided by Prof. Jeffrey A. Johnson (University of Wisconsin-Madison, WI, USA). The following oligonucleotides were used (core sequence is underlined, and mutated bases are in bold): wild-type ARE, 5′-CTC AGC CTT CCA AAT CGC AGT CAC AGT GAC TCA GCA GAA TC-3′; and GC mutant ARE, 5′-CTC AGC CTT CCA AAT CGC AGT CAC AGT GAC TCA ATA GAA TC-3′. siRNAs directed for NRF2 and HO-1 were produced from Dharmacon, Inc. (Lafayette, CO, USA).

### 2.2. Human Tissue Samples

Human breast tumor tissues were obtained from the Biorepository Lab of Breast Cancer Biology at the Cancer Research Institute, Seoul National University. The research use of these specimens was approved by the Institutional Review Board (IRB) of Seoul National University Hospital (IRB No., 1405-088-580). Tumor tissue specimens were represented different tumor stages (stage 1 (n = 2), stage 2 (n = 5), stage 3 (n = 9), stage 4 (n = 2), and unknown (n = 1)) according to the TNM classification [31]. Adjacent normal tissue specimens were obtained from different tumor stages (stage 1 (n = 2), stage 2 (n = 9), and stage 3 (n = 4)).

### 2.3. Immunohistochemistry

The human breast cancer (n = 18) and adjacent normal (n = 15) tissues were subjected to immunohistochemical analysis of COX-2 and NRF2 localization. Formalin-fixed breast samples were embedded in paraffin and sliced at 5-µm thickness, followed by staining with hematoxylin and eosin (H&E) by standard procedures. Immunohistochemistry was conducted by using Vectastain ABC kit (Vectastain, PK6101, and PK6102). Briefly, sections were deparaffinized, hydrated, and blocked with 3% hydrogen peroxide for 15 min. Then, specimens were subjected to antigen retrieval by immersing the slides in 0.01 M boiling citrate buffer, heating them in a microwave oven for 5 min, and cooling them down at room temperature. After treatment with blocking solution for 1 h, the sections were incubated with 1:25 to 1000 dilutions of appropriate antibodies at room temperature for 60 min in Tris-buffered saline containing 0.05% Tween-20. The slides were washed three times for 5 min with phosphate-buffered saline (PBS) and incubated in biotinylated anti-mouse or anti-rabbit secondary antibody for an additional 30 min at room temperature. Slides were then rinsed and incubated with avidin-biotinylated horseradish peroxidase (HRP) complex for 30 min at 37 °C. Slides were washed three times for 5 min, and COX-2 and NRF2 were visualized after incubation for 30 sec in a solution containing 3,3′-diaminobenzidine tetrahydrochloride. Hematoxylin was used as a nuclear counterstain in tissue sections. Stained slides were dehydrated through a graded series of alcohol washes and mounted using cover slides. Scoring was performed by the first author, who was blinded as to the primary antibodies and the treatment groups. For COX-2 and NRF2, the percentage of positively stained cells was estimated. Cases with 5% or less positively stained cells were scored as 0, 1 for 5–20%, 2 for 20–60%, and 3 for 60% or higher. Staining intensity was graded as absent (0), weak (1+), medium (2+), or strong (3+).

### 2.4. Measurement of 15d-PGJ_2_

The human breast cancer (n = 18) and adjacent normal (n = 13) tissues were homogenized with ice-cold PBS and centrifuged for 10 min at 3000× *g*. The diluted supernatant was applied to a preactivated Amprep^TM^ C-18 reverse phase cartridge (Amersham Pharmacia Biotech Inc.; Buckinghamshire, UK), and eicosanoids were released by ethylacetate containing 1% methanol. The extract was evaporated to dryness under a stream of nitrogen and resuspended in enzyme immunoassay buffer. The amounts of 15d-PGJ_2_ were measured by using the 15d-PGJ_2_ enzyme-immunoassay (EIA) kit (Assay Designs, Inc.; Ann Arber, MI, USA) according to the manufacturer’s protocol.

### 2.5. Cell Culture

MCF-7 cells were maintained routinely in RPMI 1640 medium supplemented with 10% FBS and a 100 ng/mL penicillin/streptomycin/fungizone mixture at 37 °C in a humidified atmosphere of 5% CO_2_/95% air. The cells were plated at an appropriate density according to each experimental scale.

### 2.6. Preparation of Nuclear Proteins

After treatment with 15d-PGJ_2_, cells (3 × 10^6^ cells/10 mL in 100-mm dish) were washed with PBS, centrifuged, and resuspended in ice-cold isotonic buffer A (10 mM HEPES, pH 7.9, 1.5 mM MgCl_2_, 10 mM KCl, 0.5 mM dithiothreitiol (DTT), and 0.2 mM phenylmethylsulfonyl fluoride (PMSF)). After the incubation in ice bath for 10 min, cells were centrifuged again and resuspended in ice-cold buffer C containing 20 mM HEPES (pH 7.9), 20% glycerol, 420 mM NaCl, 1.5 mM MgCl_2_, 0.2 mM EDTA, 0.5 mM DTT, and 0.2 mM PMSF followed by incubation at 0 °C for 20 min. After vortex-mixing, the resulting suspension was centrifuged, and the supernatant was stored at −70 °C after determination of protein concentrations.

### 2.7. Western Blot Analysis

MCF-7 cells (2 × 10^5^ cells/mL) were plated in a 60 mm dish and treated with 15d-PGJ_2_ under specified conditions. After rinsed with PBS, the cells were exposed to the lysis buffer with protease inhibitors (Cell Signaling Techology; Beverly, MA, USA) on ice for 15 min. After centrifugation at 12,000× *g* for 15 min, supernatant was separated and stored at −70 °C until use. The protein concentration was determined by using the Pierce^TM^ BCA protein assay kit (Thermo Fisher Scientific; Waltham, MA, USA) in accordance with the manufacturer’s instructions. The proteins were loaded onto a 10% sodium dodecyl sulfate polyacrylamide gel electrophoresis (SDS-PAGE) and transferred to polyvinylidene fluoride membranes (Millipore; Burlington, MA, USA). After transfer, membranes were blocked with 3% bovine serum albumin in Tris-buffered saline with 0.05% Tween 20 (TBS-T) and probed with the specified primary antibodies (diluted 1:1000) overnight at 4 °C. The membranes were washed and incubated with the appropriate secondary antibodies in TBS-T for 1 h. Blots were then developed using an enhanced chemiluminescence system (Thermo Fisher Scientific; Waltham, MA, USA).

### 2.8. Electrophoretic Mobility Shift Assay (EMSA) for Measuring Human NRF2-ARE Binding Activity

Synthetic double-strand oligonucleotide containing the NRF2 binding domain (ARE) was labeled with [γ-^32^P]ATP using T4 polynucleotide kinase and separated from un-incorporated [γ-^32^P]ATP by gel filtration using a nick spin column (Amersham Biosciences; Buckinghamshire, UK). The sequences of oligonucleotides in double strands used in the present study were 5′-TTT TCT GCT GAG TCA AGG TCC G-3′ and 3′-AAA AGA CGA CTC AGT TCC AGG C-5′. The oligonucleotide was synthesized by Bionics (Seoul, South Korea). Prior to addition of the ^32^P-labeled oligonucleotide (100,000 cpm), 10 μg of the nuclear extract was incubated on ice for 15 min in gel-shift binding buffer (4% glycerol, 1 mM EDTA, 1 mM DTT, 100 mM NaCl, 10 mM Tris-HCl (pH 7.5), and 0.1 mg/mL sonicated salmon sperm DNA). DNA-protein complexes were resolved by 6% non-denaturating polyacrylamide gel at 150 V for 2 h followed by autoradiography.

### 2.9. Immunofluorescent Analysis

MCF-7 cells were plated on the chamber slide and treated with 15d-PGJ_2_. After fixation with paraformaldehyde, samples were incubated with blocking agents (0.1% Tween-20 in PBS containing 5% bovine serum albumin), washed with PBS and then incubated with a diluted (1:100) primary antibody for overnight. After washing with PBS, samples were incubated with a FITC-conjugated secondary antibody for 1 h. Cells were also stained with propidium iodide (PI) or 4′,6-diamidino-2-phenylindole (DAPI) and examined under a confocal microscope.

### 2.10. Transient Transfection and the Luciferase Reporter Gene Assay

For the comparison of transcriptional activity of NRF2, MCF-7 cells were plated at a confluence of 60% in 6-well plate and grown in RPMI supplemented with 10% heat-inactivated FBS at 37 °C in a humidified atmosphere of 5% CO_2_/95% air. Transient transfections were performed using Lipofectamine^®^ 2000 Transfection Reagent (Invitrogen; Waltham, MA, USA) following the manufacturer’s instructions. After a 24 h transfection with plasmid (ARE-Luc) harboring the ARE binding site-luciferase construct, cells were treated with 15d-PGJ_2_ for an additional 12 h, and the cell lysis was carried out with the reporter lysis buffer. After mixing the cell extract with a luciferase substrate (Promega; Madison, WI, USA), the luciferase activity was measured by the EG&G Berthold AntoLumat LB 953 luminometer. The β-galactosidase assay was done using the promega β-Galactosidase enzyme assay system for normalizing the luciferase activity. For the measurement of VEGF promoter activity, MCF-7 cells were transfected with nonspecific siRNA or Nrf2 siRNA. After 24 h of incubation, the cells were transfected with the pVEGF luciferase reporter gene and treated with 15d-PGJ_2_ for additional 24 h. Cells were lysed as described above.

### 2.11. Chromatin Immunoprecipitation (ChIP) Assay

The ChIP assay was performed based on a protocol previously described [32]. Genomic DNA (100 ng) was immunoprecipitated with 5 μg of specific NRF2 antibody or normal mouse IgG. The primer sequence was as follows: ARE in human HO-1 promoter (F: 5′-CCC TGC TGA GTA ATC CTT TCC CGA-3′ and R: 5′-ATG TCC CGA CTC CAG ACT CCA-3′) [33].

### 2.12. Enzyme-Linked Immunosorbent Assay (ELISA)

To determine VEGF secretion levels, MCF-7 cells were plated in 60 mm culture dishes at a density of 2 × 10^5^ cells and incubated for 24 h. They were then cultured for an additional 36 h with 15d-PGJ_2_, and the culture supernatant was collected and analyzed using a VEGF Quantikine ELISA kit (cat# DVE00, R&D Systems; Minneapolis, MN, USA) following manufacturer’s instructions.

### 2.13. Aortic Ring Assay

Aortas were harvested from 6-week-old male Sprague Dawley rats, 12-week-old B6SJL NRF2^+/+^ wild type (WT), and NRF2^-/-^ knockout (KO) mice. Plates (48 well) were coated with 100 μL/well of ice-cold matrigel, and after it changed to a gel, the rings were placed in the wells and sealed in place with an overlay of 50 μL/well of matrigel. Next, 15d-PGJ_2_ was added to the wells at a final concentration of 1 μM in human endothelial serum-free medium (Invitrogen; Carlsbad, CA, USA). The assay was scored, in a double-blind manner, from 0 (least positive) to 5 (most positive). Each data point was assayed in sextuplets.

### 2.14. Ab Initio Calculation

All calculations were performed with the Hartree–Fock method, which is the first approximation and least expensive of the ab initio methods, on Gaussian 98 suite of programs at the Bioinformatics and Molecular Design Research Center in Yonsei Univeristy, Korea. The binding energy of each molecule was calculated as follows:

$$\Delta E_{bind}^{X}=E_{Complex}^{X}-(E^{CH_{3}S^{-}-}+E^{X})$$

(X = 1,2; 1 = 15d-PGJ_2_, 2 = 9,10-dihydro-15-PGJ_2_)

where *E^X^* and $E^{CH_{3}S^{-}}$ are the individual energies of 15d-PGJ_2_ or 9,10-dihydro-15d-PGJ_2_, and cysteine residues in the proteins, respectively. $\Delta E_{bind}^{1}$ and $\Delta E_{bind}^{2}$ are the binding energies of 15d-PGJ_2_ and 9,10-dihydro-15d-PGJ_2_, respectively, to the thiol residues.

### 2.15. Immunoprecipitation

The binding of biotinylated-15d-PGJ_2_ or biotinylated-9,10-dihydro-15d-PGJ_2_ to Keap1 in MCF-7 cells was examined by an immunoprecipitation assay according to the protocol described elsewhere [34]. Briefly, cellular proteins (200 µg) were subjected to immunoprecipitation by shaking with Keap1 (Santa Cruz Biotechnology; Santa Cruz, CA, USA) primary antibody at 4 °C for 12 h followed by the addition of protein G-agarose bead suspension (25% slurry, 40 µl) and shaking for 2 h. After centrifugation at 7000× *g* for 2 min, immunoprecipitated beads were collected by discarding the supernatant and rinsed with cell lysis buffer. After the final wash, immunoprecipitate was resuspended in 40 µl of 2× SDS electrophoresis sample buffer and boiled for 5 min. Supernatant (30 µl) from each sample was collected after centrifugation and loaded on SDS-polyacrylamide gel (0.75 mm thickness). After electrophoresis, separated proteins were transferred from gel to a PVDF membrane, which was then immunoblotted with HRP-conjugated streptavidin antibody (Pierce Biotechnology, Inc.; Rockford, IL, USA) to detect the interaction of biotinylated-15d-PGJ_2_ or biotinylated-9,10-dihydro-15d-PGJ_2_ with Keap1.

### 2.16. Tube Formation Assay

Each 48-well plate was coated with 150 μL/well of ice-cold Matrigel and incubated at 37 °C for 30 min. Human umbilical vein endothelial cells (HUVECs) seeded at a density of 5 × 10^4^ cells/well were grown in the 500 μL conditioned media with 10% FBS. After a 16 h incubation, the cells were photographed.

### 2.17. RNA Isolation and Reverse Transcription Polymerase Chain Reaction (RT-PCR)

Total RNA was isolated from MCF-7 cells using TRIzol^®^ reagent (Invitrogen; Carlsbad, CA, USA) according to the manufacturer’s protocol. One µg of total RNA was reverse transcribed with a Moloney murine leukemia virus reverse transcriptase (Promega; Madison, WI, USA) at 42 °C for 50 min and at 72 °C for 15 min. One μL of cDNA was amplified in sequential reactions: 95 °C for 1 min, 60 °C for 1 min, and 72 °C for 1 min, for 32 cycles of *NRF2*; 95 °C for 1 min, 60 °C for 1 min, and 72 °C for 1 min, for 25 cycles of *HO-1*; 95 °C for 1 min, 60 °C for 2 min, and 72 °C for 1 min for 30 cycles of *VEGF*; and 94 °C for 1 min, 56 °C for 2 min, and 72 °C for 2 min, for 26 cycles of the house keeping gene, *glyceraldehyde-3-phosphate dehydrogenase* (*GAPDH*), followed by a final extension at 72 °C for 10 min. The primers used for the RT-PCR reactions are as follows (forward and reverse, respectively): *HO-1*, 5′-CAG GCA GAG AAT GCT GAG TTC-3′ and 5′-GAT GTT GAG CAG GAA CGC AGT-3′; *NRF2*, 5′-CGG TAT GCA ACA GGA CAT TG-3’ and 5′-ACT GGT TGG GGT CTT CTG TG-3′; *VEGF*, 5′GAG AAT TCG GCC TCC GAA ACC ATG AAC TTT CTG T-3′ and 5′-GAG CAT GCC CTC CTG CCC GGC TCA CCG C-3′; and *GAPDH*, 5′-AAG GTC GGA GTC AAC GGA TTT-3′ and 5′-GCA GTG AGG GTC TCT CTC CT-3′. Amplification products were analyzed in 1.0% agarose gel electrophoresis, stained with ethidium bromide, and photographed under ultraviolet light.

### 2.18. Measurement of HO Activity

Confluent cells in 100 mm culture dishes were incubated for 24 h in the presence or absence of 15d-PGJ_2_ (30 µM). HO activity was determined according to the method described by Motterlini et al. [35]. HO activity was measured as picomoles of bilirubin formed per milligram of epithelial cell protein per hour. Basal HO activity was in a range between 100 pmole and 200 pmole bilirubin/mg protein/h. The enzyme activity was determined spectrophotometrically with MCF-7 cell lysates incubated for 1 h in the dark at 37 °C in the presence of hemin (10 µM), NADPH (20 µM), and 1 mg of protein from mouse liver cytosol as a source of biliverdin reductase. Reactions were terminated by adding 1 mL of chloroform, and the concentration of bilirubin was determined from the difference in absorbance between 464 nm and 530 nm using an extinction coefficient of 40 mM^−1^cm^−1^ for bilirubin.

### 2.19. Illumina Gene Expression Microarray and Data Analysis

The Illumina Gene Expression 48K microarray analysis was performed at Macrogen (Seoul, South Korea). MCF-7 cells were treated with 30 μM each of 15d-PGJ_2_ or 9,10-dihydro-15d-PGJ_2_ for 12 h in four independent experiments. The biotinylated cRNA samples for hybridization on Illumina Sentrix HumanRef-8 Expression BeadChip arrays (Illumina, Inc.; San Diego, CA, USA) were prepared according to Illumina Amplification Kit (Ambion, Inc.; San Diego, CA, USA). QIAGEN RNeasy Mini kit (Qiagen; Valencia, CA, USA) was used for cRNA purification, according to the manufacture’s recommendations. The column-purified cRNA was quality controlled using the mRNA nanochip assay on an Agilent 2100 Bioanalyzer and spectrophotometrically quantified (NanoDrop). After hybridization, the Sentrix BeadChips were washed according to Illumina’s recommended manual. BeadChips were collected by centrifugation and dried immediately. Arrays were scanned using the Illumina Bead Array Reader Scanner. Focused Illumina microarray quality was determined by image viewing and incorporated control bead analysis (housekeeping, hybridization, signal generation, and background). Normalization of all microarrays over all gene signal intensities based on the arithmetic mean was performed; consequently, 16,694 probes (detection *p* value < 0.05) were analyzed.

The samples were categorized as either molecular function or biological process and further subcategorized with the panther classification system (http://www.pantherdb.org (accessed on 2 January 2021)). A hierarchical clustering (Euclidean method and complete linkage) was then performed on samples and genes. The statistical analysis for single comparison was performed using the Avadis Prophetic version.3.3 (Strand Genomics, Bangalore, India) and R (version 2.4.0).

### 2.20. The Cancer Genome Atlas (TCGA) Data Analysis

The Cancer Genome Atlas for breast cancer (TCGA-BRCA) RNAseqV2 gene expression data and clinical data were obtained from the TCGA data portal (https://portal.gdc.cancer.gov (accessed on 2 January 2021)) in October 2020. Altogether, 1075 breast cancers from TCGA with normalized gene expression and specific clinical status were collected and analyzed (up to October 2020). The relationship between the expressions of genes in breast cancer was analyzed using this database. Using OncoLnc (http://www.oncolnc.org/ (accessed on 2 January 2021)) platform, Cox-regression-analysis data were acquired for *COX-2*, *NRF2,* and *HO-1* in breast cancers. These data were then used to generate Kaplan–Meier plots. Kaplan–Meier survival analysis was performed to compare the survival distribution between different groups by using GraphPad Prism software (Version 8.0, GraphPad software Inc., San Diego, CA, USA) [36]. A plot of the Kaplan–Meier analysis with appropriate sample size provides the information on the length of survival, median survival time of the distinct sample populations, and significance of the difference between the survival curves.

### 2.21. Statistical Analysis

All data are expressed as means ± standard deviation (SD). Each experiment was performed a minimum of three times. Statistical analysis of was performed using one-way analysis of variance (ANOVA). Student’s *t*-test was used to determine differences regarding the expression of COX-2 and NRF2 between adjacent normal tissues and breast cancer tissues. Statistical significance was accepted at *p* < 0.05.

## 3. Results

### 3.1. COX-2 Expression and 15-Deoxy-Δ^12,14^-prostaglandin J_2_ Production Are Elevated in Human Breast Cancer

**Figure 1:**
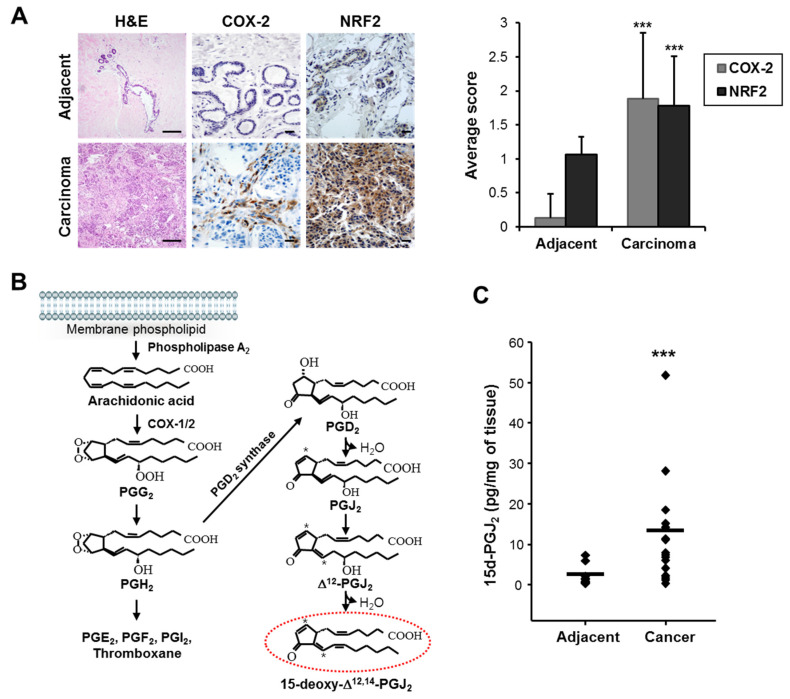
The expression levels of cyclooxygenase-2 (COX-2) and nuclear factor erythroid 2-related factor 2 (NRF2) and 15-deoxy-Δ^12,14^-prostaglandin J_2_ (15d-PGJ_2_) production are increased in human breast cancer tissues. (**A**) The expression of COX-2 and NRF2 in human breast cancer tissues vs. surrounding normal tissues. Paraffin-embedded human breast cancer or adjacent normal tissues were immunostained for COX-2 and NRF2 and counterstained with hematoxylin, as described in the Materials and Methods section. Scale bars indicate 100 μm. (**B**) Formation of 15d-PGJ_2_. Phospholipase A_2_ catalyzes hydrolytic release of arachidonic acid (AA) from membrane phospholipids. Cyclooxygenases catalyze oxidative conversion of arachidonic acid (AA) to PGH_2_. PGD_2_, a precursor of 15d-PGJ_2_, is formed by the action of PGD_2_ synthase. Alternatively, other prostaglandins (e.g., PGE_2_, PGF_2α_, and PGI_2_) and thromboxanes are formed. PGD_2_ undergoes chemical dehydration to form the PGJ_2_. PGJ_2_ is then subjected to further dehydration by loss of the 15-hydroxyl group, which, coupled with migration of the 13,14-double bond of PGJ_2_, results in the formation of 15d-PGJ_2_. Asterisks indicate the positions of the chemically reactive electrophilic carbon center. (**C**) The elevated levels of 15d-PGJ_2_ in human breast cancer tissues. 15d-PGJ_2_ production in human breast cancer and adjacent normal tissues was measured as described in the Materials and Methods section. Data are means ± standard deviation (SD). *** *p* < 0.001, significantly different compared to adjacent normal breast tissues.

It is well known that COX-2, a rate-limiting enzyme in the biosynthesis of prostaglandins, is overexpressed constitutively in various cancer cells and recognized as one of the hallmarks of chronic inflammation-associated malignancies. COX-2 has been shown to contribute to carcinogenesis by promoting cell proliferation and angiogenesis as well as by protecting cells from apoptosis (reviewed in [37], and see references therein). Immunohistochemical analysis revealed that the expression levels of COX-2 and the stress-responsive transcription factor NRF2 were significantly increased in breast tumor tissues (n = 18) compared to adjacent normal tissues (n = 15) (Figure 1A). Details are shown in Supplementary Table S1. 15d-PGJ_2_ is one of the major terminal products of COX-2 (Figure 1B). As shown in Figure 1C, tumors (n = 18) exhibited high levels of 15d-PGJ_2_ compared to adjacent normal tissues (n = 13). 

### 3.2. 15d-PGJ_2_ Increases the Nuclear Accumulation of NRF2 and Its Binding to ARE 

**Figure 2:**
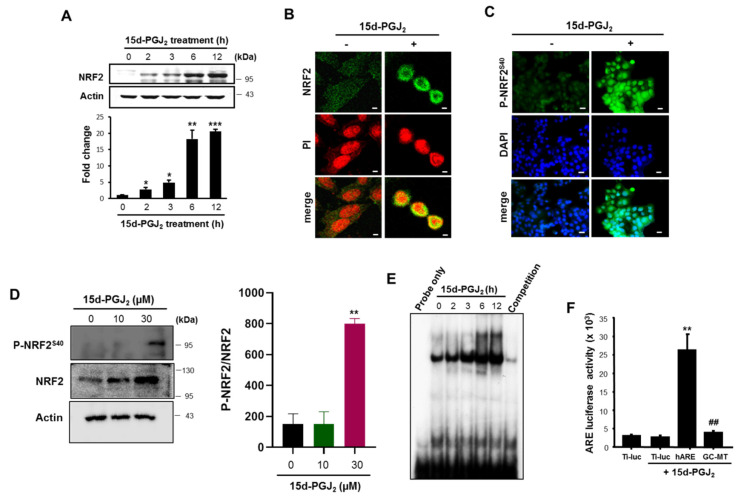
15d-PGJ_2_ induces nuclear translocation and antioxidant responsive element (ARE) binding of NRF2. (**A**) The level of nuclear NRF2 was assessed by Western blot analysis. Data are means ± SD. *p < 0.05, **p < 0.01, and ***p < 0.001, significantly different compared to control. (**B**) Immunocytochemical analysis was performed using anti-NRF2 antibody after the treatment of MCF-7 cells with 30 µM 15d-PGJ_2_ for 12 h. Cells were stained with propidium iodide (PI) and visualized by confocal microscopy. Scale bars indicate 50 μm. **(C**) Immunocytochemical detection of phosphorylated Nrf2 in MCF-7 cells with 30 µM 15d-PGJ_2_ for 24 h. Cells were also stained with 4′,6-diamidino-2-phenylindole (DAPI) to identify the nuclei (blue). Scale bars indicate 200 μm. (**D**) The phosphorylation of NRF2 was assessed by Western blot analysis. The experimental conditions are same as (C). (**E**) The ARE binding activity was measured by electrophoretic mobility shift assay (EMSA). Nuclear extracts were isolated after treatment with 30 µM 15d-PGJ_2_ for indicated time periods. (**F**) For the comparison of transcriptional activity of NRF2, cells were transiently transfected with plasmid (ARE-Luc) harboring the ARE-binding site-luciferase construct, GC mutant ARE (GC-MT), or control vector (Ti-Luc). After overnight transfection, cells were exposed to 30 µM 15d-PGJ_2_ for 12 h and treated with reporter lysis buffer for the measurement of the luciferase activity. ** *p* < 0.01, significantly different from empty vector-transfected control; ##*p* < 0.01, significantly different compared to hARE-transfected control.

To determine the association between COX-2 and NRF2 overexpressed in human breast tumor, we examined the effects of 15d-PGJ_2_, one of the major products of COX-2 on the activation of NRF2. As illustrated in Figure 2A,B, nuclear accumulation of NRF2 was evident in MCF-7 cells treated with 30 μM of 15d-PGJ_2_. NRF2 is phosphorylated on the serine 40 residue by some kinases, and this facilitates its translocation into nucleus. We found that 15d-PGJ_2_ treatment markedly enhanced the accumulation of phosphorylated NRF2 (P-NRF2), predominantly in the nucleus as assessed by immunocytochemical (Figure 2C) and Western blot (Figure 2D) analyses. The ARE-binding activity of NRF2 induced by 15d-PGJ_2_ was increased in a time-dependent manner as assessed by EMSA (Figure 2E). The specificity of ARE-binding activity of NRF2 was verified in a competition assay using an excess of unlabeled ARE-oligo (Figure 2E, last lane). Furthermore, the cells stimulated with 15d-PGJ_2_ exhibited an enhanced ARE luciferase activity with more than eight-fold induction (Figure 2F). However, mutation of the ARE core sequence (GC-box) markedly reduced the ARE luciferase activity.

### 3.3. 15d-PGJ_2_ Induces VEGF Expression and Angiogenesis through NRF2 Activation

**Figure 3:**
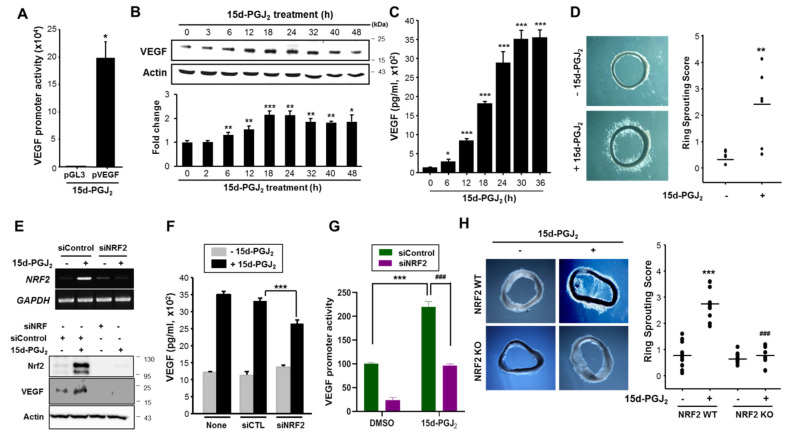
NRF2 mediates 15d-PGJ_2_-induced vascular endothelial growth factor (VEGF) expression and angiogenesis. (**A**) The transcriptional activity of VEGF was measured by the luciferase reporter gene assay. After transfection with the VEGF-luciferase construct, MCF-7 cells were treated with 15d-PGJ_2_ (30 μM) for 12 h. * *p* < 0.05, significantly different compared to empty vector-transfected control. (**B**) MCF-7 cells were treated with 30 µM of 15d-PGJ_2_ and incubated for indicated time periods. Protein extracts were separated by sodium dodecyl sulfate polyacrylamide gel electrophoresis (SDS-PAGE), and Western blot analysis was conducted for detecting the expression of VEGF protein. Data are means ± SD. * *p* < 0.05, ** *p* < 0.01, and *** *p* < 0.001, significantly different compared to control. (**C**) An enzyme immunoassay was conducted to measure VEGF production by 15d-PGJ_2_ in MCF-7 cells. * *p* < 0.05 and *** *p* < 0.001, significantly different from vehicle control. (**D**) Aortic segments were harvested from SD rats (n = 6 per group). Aorta in matrigel was treated with 1 μM of 15d-PGJ_2_ for 5 days. Endothelial-cell sprouts forming branching cords from the margins of vessel segments taken from rat were photographed under a phase-contrast microscope. Sprouting scores were classified from 0 (least positive) to 5 (most positive). Data are means ± SD. ** *p* < 0.01, significantly different compared to vehicle control. (**E,F**) MCF-7 cells were transfected with NRF2 siRNA for 24 h and then incubated with 15d-PGJ_2_ for an additional 24 h. The expression (E) and production (F) of VEGF were measured by Western blot analysis and enzyme-linked immunosorbent assay (ELISA), respectively. Data are means ± SD. *** *p* < 0.001, significantly different compared to nonspecific siRNA-transfected control. (**G**) After transfection of MCF-7 cells with control siRNA or siNRF2, the VEGF promoter activity was determined by the luciferase reporter gene assay as described in the Materials and Methods section. *** *p* < 0.001, significantly differrent from vehicle control; ### *p* < 0.001, significantly different compared to the 15d-PGJ_2_-treated control siRNA-transfected cells. (**H**) Aortic segments were harvested from NRF2^-/-^ KO and NRF2^+/+^ wild-type (WT) mice (n = 10 per group). Aortas in Matrigel were treated with 1 μM of 15d-PGJ_2_ for 5 days. Endothelial-cell sprouts forming branching cords from the margins of vessel segments taken from mice were photographed under a phase-contrast microscope. Sprouting scores were classified from 0 (least positive) to 5 (most positive). Data are means ± SD. *** *p* < 0.001, significantly different from vehicle control; ###*p* < 0.001, significantly different compared to the 15d-PGJ_2_-treated NRF2 WT group.

VEGF, one of the major target genes of hypoxia inducible factor (HIF)-1, specifically recruits endothelial cells into hypoxic and vascular areas and stimulates their proliferation. The representative transcription-factor recognition site located in the VEGF promoter is HRE, which is a preferential binding site of HIF-1 under hypoxia. To confirm the effect of 15d-PGJ_2_ on VEGF promoter activity, we performed the luciferase reporter gene assay. In the MCF-7 cells treated with 15d-PGJ_2_, the VEGF promoter activity was increased by approximately 20-fold (Figure 3A). As a result, VEGF protein expression was enhanced as measured by Western blot analysis (Figure 3B). Furthermore, there was a significant time-dependent escalation of VEGF release (*p* < 0.001) in the medium of MCF-7 cells after stimulation with 15d-PGJ_2_ (Figure 3C). Next, we examined whether the elevated levels of VEGF induced by 15d-PGJ_2_ could contribute to angiogenesis. As illustrated in Figure 3D, 15d-PGJ_2_ treatment significantly stimulated vessel sprouting.

Multiple lines of evidence support that elevated NRF2 activity may play a role in the evolution of cancer [30]. 15d-PGJ_2_-induced expression and promoter activity of VEGF as well as NRF2 activation prompted us to determine whether NRF2 signaling is important for angiogenic activities of 15d-PGJ_2_. Upon knockdown of NRF2 expression by employing the NRF2 siRNA, the 15d-PGJ_2_-induced VEGF expression (Figure 3E) and production (Figure 3F) were diminished substantially. As shown in Figure 3G, transfection with NRF2 siRNA abolished 15d-PGJ_2_-induced expression of VEGF promoter activity. These results indicate that NRF2 is involved in 15d-PGJ_2_-induced angiogenesis. To further assess the role of NRF2 in 15d-PGJ_2_-induced angiogenesis, we compared endothelial-cell sprouting in the aortic ring segments of NRF2^-/-^ and NRF2^+/+^ mice. Notably, 15d-PGJ_2_ approximately induced a three-fold increase in endothelial-cell sprouting in the WT mice, whereas this effect was significantly abrogated in the NRF2^-/-^ mice (Figure 3H). Taken together, these results suggest that NRF2 plays a crucial role in mediating 15d-PGJ_2_-induced angiogenesis through VEGF upregulation.

### 3.4. The α,β-Unsaturated Carbonyl Moiety Present in the Cyclopentenone Ring of 15d-PGJ_2_ Is Essential for Its Induction of NRF2 Activation, VEGF Upregulation, and Angiogenesis

The presence of the α,β-unsaturated carbonyl group in the cyclopentenone ring of 15d-PGJ_2_ has been suggested to be prerequisite for its alteration of cellular redox status and/or the modulation of target protein functions [38]. This unique chemical property of 15d-PGJ_2_ allows it to form covalent adducts with free thiols in glutathione or in several proteins by Michael addition [38]. NRF2 is sequestered in the cytoplasm by the inhibitory protein Keap1. The oxidation or covalent modification of some sensor cysteine residues of Keap1 facilitates its release of NRF2 for nuclear translocation.

**Figure 4:**
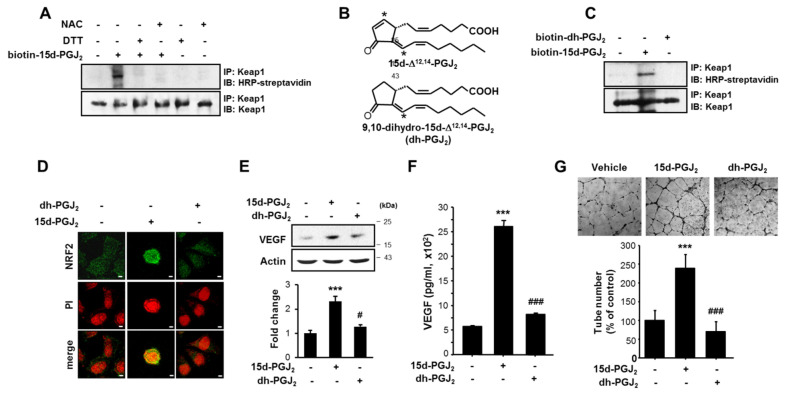
The α,β-unsaturated carbonyl moiety of 15d-PGJ_2_ is essential for the activation of NRF2, expression/production of VEGF, and further angiogenesis induced by this cyclopentenone prostaglandin. (**A**) MCF-7 cells were incubated with 30 μM of biotinylated 15d-PGJ_2_ in the presence of dithiothreitiol (DTT) (1 mM) and N-acetylcysteine (NAC) (5 mM) for 9 h. The interaction between Keap1 and 15d-PGJ_2_ was assessed by immunoblot analysis, and the incorporation of biotinylated 15d-PGJ_2_ into Keap1 immunoprecipitates was detected with horseradish peroxidase (HRP)-streptavidin and enhanced chemiluminescence (ECL). (**B**) The chemical structures of 15d-PGJ_2_ and biotinylated 9,10-dihydro-15d-PGJ_2_ (dh-PGJ_2_). Asterisks depict electrophilic carbons (position 9 and 13). (**C**) MCF-7 cells were incubated with 30 μM of biotinylated 15d-PGJ_2_ or biotinylated dh-PGJ_2_. The interaction between Keap1 and 15d-PGJ_2_ or dh-PGJ_2_ was assessed by immunoblot analysis with HRP-streptavidin and ECL. (**D**) Immunocytochemical analysis was performed using anti-NRF2 antibody after the treatment of MCF-7 cells with 30 µM each of 15d-PGJ_2_ and dh-PGJ_2_ for 12 h. Scale bars indicate 50 μm. Western blot analysis (**E**) and ELISA (**F**) and were performed to examine the levels of VEGF in MCF-7 cell treated with 15d-PGJ_2_ or dh-PGJ_2_. Data are means ± SD. *** *p* < 0.001, significantly different compared to vehicle control; # *p* < 0.05 and ### *p* < 0.001, significantly different compared to 15d-PGJ_2_-treated cells. (**G**) HUVECs were treated with various conditioned media (CM) with 10% fetal bovine serum (FBS). After 16 h incubation with 15d-PGJ_2_ and dh-PGJ_2_, microphotographs were taken 40×. Representative endothelial tubes were shown. Tube formation (%) was quantified by the number of three-way branching point. Values are means ± SD of three independent experiments. *** *p* < 0.001, significantly different from vehicle control; ### *p* < 0.001, significantly different compared to 15d-PGJ_2_-treated cells.

To examine whether the 15d-PGJ_2_-induced NRF2 activation is associated with its direct interaction with Keap1, biotin-conjugated 15d-PGJ_2_ was utilized. As shown in Figure 4A, the binding of biotinylated 15d-PGJ_2_ to Keap1 was confirmed by immunoprecipitation, which was completely suppressed by the treatment with N-acetylcysteine (NAC) and DTT, well-known thiol modification agents (Figure 4A).

To determine whether the activation of pro-angiogenic signaling by 15d-PGJ_2_ is attributed to its electrophilic α,β-unsaturated carbonyl functional group, we utilized 9,10-dihydro-15d-PGJ_2_ that lacks the cyclopentenone moiety (Figure 4B). For this purpose, we initially estimated the binding energy for interaction between 15d-PGJ_2_ and thiol residues in cellular proteins using *ab initio* transport calculations. Table S2 shows that $\Delta E_{bind}^{1}$ for 15d-PGJ_2_ is 0.06 kcal/mol and for 9,10-dihydro-15d-PGJ_2_ is $\Delta E_{bind}^{2}$ is 4.27 kcal/mol. These results suggest that the binding of 15d-PGJ_2_ to the thiol residues in cellular proteins is thermodynamically more favorable than that of 9,10-dihydro-15d-PGJ_2_. In line with this estimation, biotinylated 9,10-dihydro-PGJ_2_ neither bound to Keap1 (Figure 4C) nor translocated into nucleus (Figure 4D) in MCF-7 cells. Moreover, 9,10-dihydro-15d-PGJ_2_ was ineffective in inducing expression (Figure 4E) and production (Figure 4F) of VEGF. To compare the angiogenic activity of 15d-PGJ_2_ and 9,10-dihydro-PGJ_2_, capillary formation in HUVECs was measured. In contrast to 15d-PGJ_2_, 9,10-dihydro-PGJ_2_ failed to induce the tubular growth in endothelial cells (Figure 4G). Taken together, these results suggest that the electrophilic carbon at the position 9 of 15d-PGJ_2_ is required for inducing angiogenesis by this cyclopentenone prostaglandin.

### 3.5. HO-1 Induced by 15d-PGJ_2_ Mediates VEGF Expression and Angiogenesis in Human Breast Cancer

**Figure 5:**
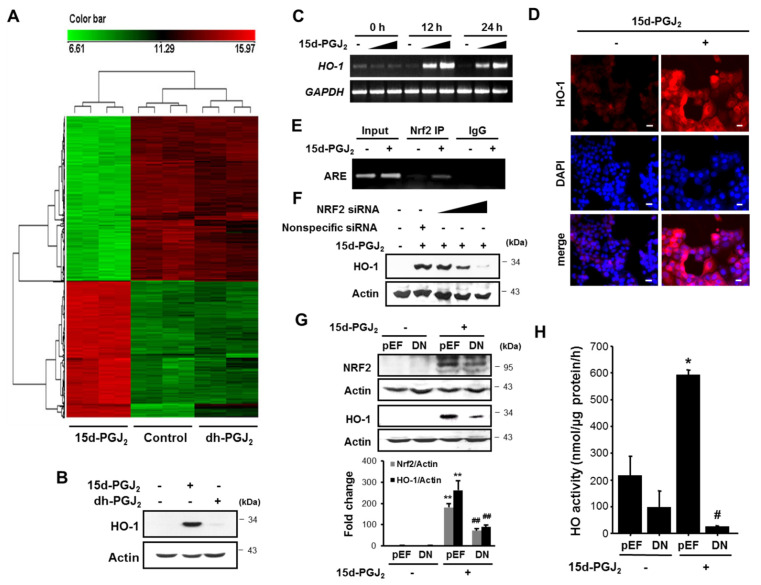
15d-PGJ_2_ induces expression/activity of HO-1 through NRF2 activation. (**A**) Cluster analysis of microarray data. Hierarchical clustering of 12 samples and 16,694 probes sets with significant variation in RNA expression levels across all samples. Overall analysis of 12 experiments carried out for microarray analysis of the gene expression profiles in MCF-7 cells after treatment with 30 μM each of 15d-PGJ_2_ or 9,10-dihydro-15d-PGJ_2_ (dh-PGJ_2_) for 12 h. Downregulated genes are represented in green (Cy3) and upregulated genes in red (Cy5). The horizontal color bar provides a visual indication of the level of changes in each PGJ_2_-treated cell vs. control. (**B**) Western blot analysis was performed to examine the expression of HO-1 protein in MCF-7 cell treated with 15d-PGJ_2_ (30 μM) or dh-PGJ_2_ (30 μM). (**C**) RT-PCR was conducted to examine the levels of the HO-1 mRNA transcript in MCF-7 cell treated with 15d-PGJ_2_ (0, 3, and 30 μM). **(D**) Immunocytochemical localization of HO-1 was analyzed after the treatment of MCF-7 cells with 30 µM 15d-PGJ_2_ for 24 h. Cells were also stained with DAPI to visualize the nuclei (blue). Scale bars indicate 200 μm. (**E**) MCF-7 cells were treated with 15d-PGJ_2_ for 12 h and harvested to determine the ARE binding activity by the chromatin immunoprecipitation (ChIP) assay. Chromatin immunoprecipitated DNA was analyzed by RT-PCR with primers for ARE element-containing region of the human HO-1 promoter. (**F**) MCF-7 cells were transfected with control or NRF2 siRNA (1, 5, and 10 μM) for 24 h and then incubated with 30 μM 15d-PGJ_2_ for an additional 24 h. The expression of HO-1 was measured by Western blotting. (**G**) MCF-7 cells were transfected with control vector (pEF) and DN-NRF2. After 24 h of incubation, cells were treated with 30 µM of 15d-PGJ_2_ for 24 h. The expression of NRF2 and HO-1 was determined by Western blot analysis. Data are means ± SD. ** *p* < 0.01, significantly different compared to vehicle control; ## *p* < 0.01, significantly different from pEF-transfected cells. (**H**) 15d-PGJ_2_-induced HO activity was determined in MCF-7 cells transfected with DN-NRF2 as well as in vector-transfected control cells. Data are means ± SD. * *p* < 0.05, significantly different compared to vehicle control; # *p* < 0.05, significantly different from empty vector-transfected cells in the presence of 15d-PGJ_2_.

For systematic comparison of differential gene expression between 15d-PGJ_2_ and its non-electrophilic analogue, we conducted microarray-based gene expression analysis. A total of 48,095 genes expressed in the MCF-7 cells were evaluated 12 h after treatment with 30 μM each of 15d-PGJ_2_ or 9,10-dihydro-PGJ_2_ (Figure 5A). Of these, 16,694 genes showed significantly different expression profiles (SAM median FDR= 0). We found enrichment in three functional groups based on the oligonucleotide annotation from the panther Classification system (Supplementary Table S3). Genes involved in heat shock response, protein folding, antioxidation and free radical removal, detoxification, stress response, cell structure and motility, and cell proliferation and differentiation were upregulated to a greater extent than what would be predicted by using a random sampling. Notably, the largest number of genes upregulated by 15d-PGJ_2_ are involved in the antioxidation, detoxification, and stress response. However, 9,10-dihydro-PGJ_2_ barely induced differential gene expression in MCF-7 cells. Expression of antioxidant enzymes increased by a maximum of approximately 90-fold (Supplementary Table S3). Notably, genes encoding antioxidant enzymes and heat-shock proteins were among the top genes that exhibited the greatest level of upregulation in MCF-7 cells treated for 12 h with 15d-PGJ_2_. Among them, *HSP32* encoding HO-1 and *HSP70B’*/*HSPA6* encoding HSP 70 were the most highly induced genes with about 90- and 86-fold increases, respectively, upon stimulation with 15d-PGJ_2_. Moreover, expression of the cell structure and motility genes increased 12- to 29-fold (Supplementary Table S3). There was a modest increase in expression of genes involved in cell proliferation and differentiation. From these findings, it is concluded that 15d-PGJ_2_ confers the survival advantage to the breast cancer cells by triggering expression of those proteins involved in antioxidant defense, detoxification, stress response, protein folding, cell structure and motility, and cell proliferation and differentiation.

Many studies on gene structures of various antioxidant enzymes have revealed the presence of the ARE/electrophile responsive element (EpRE) sequence in the promoter regions of glutathione *S*-transferase [39], NAD(P)H:quinone oxidoreductase [40,41], HO-1 [42], and glutamate cysteine ligase [43]. Thus, the ARE/EpRE sequence plays a pivotal role in the regulation of the cellular defense against oxidative stress [44]. To investigate whether the increased expression of antioxidant genes corresponded to an elevation in their protein expression levels, we performed Western blot and RT-PCR analyses in MCF-7 cells after treatment with 15d-PGJ_2_. Notably, 15d-PGJ_2_ treatment markedly enhanced the protein (Figure 5B) and mRNA (Figure 5C) levels of HO-1, a representative antioxidant enzyme, which was consistent with the microarray data. Again, 9,10-dihydro-PGJ_2_ could not induce HO-1 expression (Figure 5B). Immunofluorescence analysis also revealed that the 15d-PGJ_2_ treatment resulted in increased expression of HO-1, which was detected in both cytoplasm and nucleus (Figure 5D). Next, the ChIP assay was performed to determine whether there was an association between the activation of NRF2 by 15d-PGJ_2_ and the expression of HO-1. As shown in Figure 5E, the direct binding of NRF2 to the ARE consensus region of the HO-1 promoter was observed. We then examined the functional role of NRF2 in 15d-PGJ_2_-mediated HO-1 expression. siRNA silencing of NRF2 attenuated HO-1 expression (Figure 5F). As an alternate approach, MCF-7 cells were transfected transiently with DN-NRF2. DN-NRF2 transfection led to significant reduction of HO-1 expression (Figure 5G) and activity (Figure 5H) induced by 15d-PGJ_2_. Taken together, these results suggest that NRF2 plays a crucial role in mediating 15d-PGJ_2_-induced expression of HO-1.

**Figure 6:**
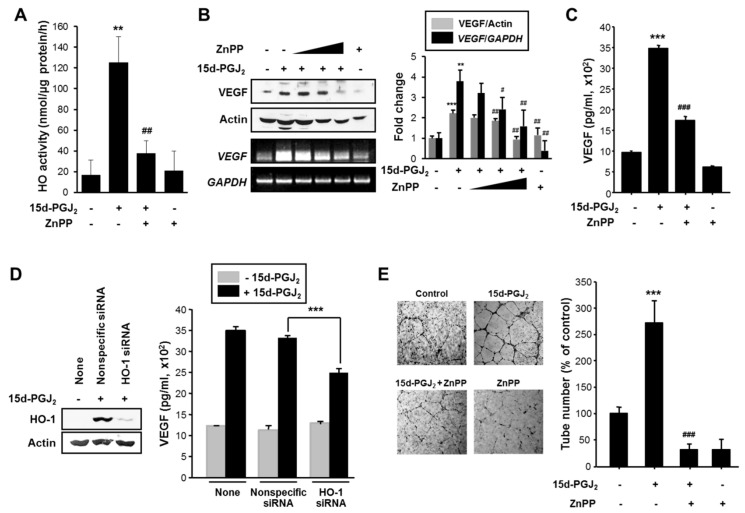
15d-PGJ_2_ induces angiogenesis through upregulation of HO-1 mediated by NRF2 activation. (**A**) 15d-PGJ_2_-induced HO activity was determined in MCF-7 cells treated with zinc protoporphyrin IX (ZnPP) (10 μM), an HO-1 inhibitor, for 24 h. Data are means ± SD. ** *p* < 0.01, significantly different compared to vehicle control; ## *p* < 0.01, significantly different from 15d-PGJ_2_-treated cells. (**B**) Treatment with ZnPP (1 5, and 20 μM) abrogated the 15d-PGJ_2_-induced expression of VEGF protein and mRNA. Data are means ± SD. ** *p* < 0.01 and *** *p* < 0.001, significantly different compared to vehicle control; # *p* < 0.05 and ## *p* < 0.01, significantly different from 15d-PGJ_2_-treated cells. (**C**) The enzyme immunoassay was conducted to measure the VEGF production in MCF-7 cells exposed to 30 µM 15d-PGJ_2_ in the presence or absence of ZnPP. Data are means ± SD. *** *p* < 0.001, significantly different compared to vehicle control; ### *p* < 0.001, significantly different from 15d-PGJ_2_-treated cells. (**D**) MCF-7 cells were transfected with HO-1 siRNA for 24 h and treated with 15d-PGJ_2_ for an additional 24 h. Western blotting was conducted to confirm HO-1 knockdown. The production of VEGF was determined by ELISA. Data are means ± SD. *** *p* < 0.001, significantly different compared to nonspecific siRNA-transfected cells. (**E**) HUVECs were treated with conditioned media containing 10% FBS. After 16 h incubation with 15d-PGJ_2_ (0 or 30 µM) in the presence or absence of 10 µM ZnPP, microphotographs were taken (’40). Representative endothelial tubes were shown. Tube formation (%) was quantified by the number of three-way branching point. Values are means ± SD of three independent experiments. *** *p* < 0.001, significantly different compared to vehicle control; ### *p* < 0.001, significantly different from 15d-PGJ_2_-treated cells.

Angiogenesis is a vital event for the growth and metastasis of tumors which is mainly regulated by VEGF. Therefore, the possible contribution of HO-1 to the expression/production of VEGF was explored. For this purpose, we utilized ZnPP, a chemical inhibitor of HO-1 activity (Figure 6A). Treatment of MCF-7 cells with 10 μM of ZnPP attenuated15d-PGJ_2_–induced HO activity as assessed by production of its product bilirubin. However, ZnPP treatment did not lower the basal HO activity. As this assay measures both HO-1 and HO-2 activities, ZnPP may not completely suppress the overall enzyme activity. Indeed, ZnPP has been shown to be least inhibitory toward HO-2 among the metal protoporphyrin HO inhibitors tested [45]. As shown in Figure 6B,C, the 15d-PGJ_2_-induced enhancement of VEGF expression and secretion was markedly reduced by ZnPP, respectively. To further verify the role of HO-1 in induction of VEGF, we transfected cells with the siRNA construct to specifically knockdown the HO-1 gene expression. Silencing of HO-1 expression substantially reduced 15d-PGJ_2_-induced VEGF production (Figure 6D). To evaluate the role of HO-1 in angiogenic activity of 15d-PGJ_2_, capillary formation in HUVECs was also measured. When HUVECs were placed on Matrigel in the presence of VEGF, there was formation of elongated and robust tube-like structures (Figure 6E). The tubular growth in cells treated with 15d-PGJ_2_ closely resembles that observed in VEGF-treated positive control cells. However, pharmacologic inhibition of HO-1 abolished the 15d-PGJ_2_-induced increase in the width and length of endothelial tubes (Figure 6E).

### 3.6. Overexpression of COX-2 and NRF2 or COX-2 and HO-1 Correlates with Poor Clinical Outcomes in Breast Cancer Patients

**Figure 7:**
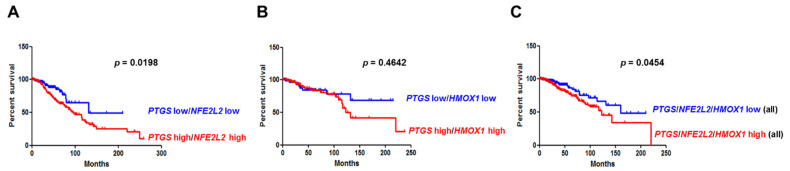
Elevated coexpression of genes encoding COX-2 and NRF2 or COX-2, NRF2 and HO-1 is associated with poor clinical outcomes. (**A**) Analysis of both *PTGS2* and *NFE2L2* expression showed breast cancer-specific survival (low expression of both *PTGS2* and *NFE2L2*, n = 142; high expression of both *COX-2* and *NFE2L2*, n = 178) from public The Cancer Genome Atlas (TCGA) data sets. (**B**) Analysis of gene encoding both COX-2 and HO-1 in relation to breast cancer-specific survival (low expression of both *PTGS2* and *HMOX1*, n = 96; high expression of both *PTGS2* and *HMOX1*, n = 109) from public TCGA data sets. (**C**) Analysis of all three *PTGS2, NFE2L2*, and *HMOX1* expression showed breast cancer-specific survival (low expression of all three *PTGS2, NFE2L2*, and *HMOX1*, n = 46; and high expression of all three *PTGS2, NFE2L2*, and *HMOX1*, n = 90) from public TCGA data sets. *PTGS2*, *NFE2L2*, and *HMOX1* denote human genes that encode COX-2, NRF2, and HO-1, respectively.

To address the clinicopathological significance of the expression of COX-2, NRF2, or HO-1, we analyzed the RNAseq results of breast cancer patient samples (N = 1075) from The Cancer Genome Atlas (TCGA) database. High expression of *PTGS2*, *NFE2L2*, and *HMOX1* alone encoding COX-2, NRF2 and HO-1, respectively, showed no significant correlation with breast cancer patients’ survival. However, coexpression of high levels of both PTGS2 and NFE2L2 was found to be significantly associated with the increased survival rate in breast cancer patients (*p* < 0.05, Figure 7A). High expression of both *PTGS2* and *HMOX1*was not statistically significant, but after about 8 years, it tended to be associated with the survival probabilities for breast cancer patients (Figure 7B). Moreover, high expression of all three genes (*PTGS2*, *NFE2L2*, and *HMOX1*) correlated with poor prognosis in breast cancer patients (Figure 7C). Taken together, these findings indicate that the expression of *PTGS2*, *NFE2L2*, and *HMOX1* correlates with poor clinical outcomes and may be exploited as diagnostic and/or therapeutic targets in breast cancer.

## 4. Discussion

A plethora of data support the role of HO-1 in the cytoprotection against a wide variety of stresses [46]. In addition to its essential role in regulating iron homeostasis, HO-1 displays potent antioxidant and anti-inflammatory functions which are mainly mediated by the heme degradation products bilirubin and CO. Notably, 15d-PGJ_2_, an anti-inflammatory prostaglandin produced at the inflamed sites, is capable of upregulating the expression of HO-1 [47,48]. The redox-sensitive transcription factor NRF2 binds to a cis-acting element known as ARE or EpRE located in the promoter region of many antioxidant and other cytoprotective genes. It is well known that expression of HO-1 is mainly regulated by NRF2 [49]. In the absence of oxidative or electrophilic stress signals, NRF2 is sequestered in the cytoplasm as an inactive complex with Keap1. Upon exposure to oxidative stress or electrophiles, NRF2 dissociates from its repressor Keap1 and translocates to the nucleus [50]. In the nucleus, NRF2 interacts with ARE/EpRE in the promoter of NRF2-responsive genes including that encodes HO-1 and stimulates their transcription. In the present work, 15d-PGJ_2_ increased the levels of NRF2 in the nucleus and its binding to ARE in MCF-7 cells.

According to Numazawa et al. [51], NRF2 can be phosphorylated by protein kinase C and subsequently translocates to the nucleus. Similarly, PI3K/Akt-mediated phosphorylation of NRF2 is likely to facilitate its dissociation from Keap1 and/or nuclear translocation in 15d-PGJ_2_-stimulated MCF-7 cells. However, it remains unclarified which of Ser/Thr residues of NRF2 can be phosphorylated by PI3K. Treatment with cycloheximide, a protein synthesis inhibitor, abrogated the nuclear accumulation of NRF2 even after 15d-PGJ_2_ treatment (data not shown). These findings suggest that besides stimulation of dissociation from Keap1 and subsequent nuclear translocation, de novo synthesis of NRF2 can also contribute to enhanced nuclear accumulation of this transcription factor provoked by 15d-PGJ_2_.

Interestingly, 15d-PGJ_2_ possesses an α,β-unsaturated carbonyl group in the cyclopentenone ring that can form covalent adducts with cysteine thiols via Michael addition [52]. This may result in the alteration of cellular redox status and/or the modulation of protein functions. It has been reported that several proteins, such as IKKβ [53], p65 [54], p50 [55], p53 [56], thioredoxin [57], c-Jun [58,59], H-Ras [60], STAT3 [61], etc. can be covalently modified by 15d-PGJ_2_. Moreover, Kim et al. [62] showed that 15d-PGJ_2_ could activate NRF2 by covalently binding to Keap1, resulting in an NRF2-dependent induction of HO-1 expression. Shiraki and colleagues [63] have shown that 15d-PGJ_2_ covalently binds to a cysteine residue in the PPARγ ligand-binding pocket through a Michael addition reaction. Therefore, NRF2 activation in MCF-7 cells by 15d-PGJ_2_ can also be achieved alternatively through covalent modification of the cysteine thiol(s) of Keap1 by the α,β-unsaturated carbonyl group present in 15d-PGJ_2_. 

In this study, the capability of 15d-PGJ_2_ to bind thiol residues in cellular proteins was estimated to be stronger than that of 9,10-dihydro-15-PGJ_2_, according to *ab initio* calculations. In addition, we have also used biotin-conjugated 15d-PGJ_2_ and biotinylated 9,10-dihydro-15d-PGJ_2_ to explore the direct interaction of these cyclopentenone prostaglandins with Keap1. The treatment with biotinylated 9,10-dihydro-PGJ_2_ did not induce the binding to Keap1, suggesting that carbon 9 in the cyclopentane ring of 15d-PGJ_2_ is critical for covalent modification of the cysteine residues in Keap1. Moreover, the treatment with NAC and DTT, well-known thiol modification agents, abrogated the binding of biotinylated 15d-PGJ_2_ to Keap1, indicative of the involvement of cysteine residue(s) in the interaction between 15d-PGJ_2_ and Keap1 proteins in MCF-7 cells.

As an enzyme involved in cellular responses to oxidative stress, HO-1 has both antitumor or protumor properties [11]. HO-1 overexpression has been reported to retard hepatocellular carcinoma progression through downregulation of several onco-micro-RNAs such as miR-30d and miR-107 [64]. Moreover, HO-1 inhibits growth of lung mucoepidermoid carcinoma in a murine xenograft model by targeting the oncogenic miR-378 and matrix metalloproteinases [65]. The effects of HO-1 induction in a mouse model of chronic liver inflammation and fibrogenesis were investigated. Administration of the HO-1 inducer, cobalt protoporphyrin IX (CoPP) ameliorated experimentally ind’uced fibrosis by regulating immune cell infiltration or proliferation as well as tumor necrosis factor receptor signaling [66]. By interfering with chronic inflammation and fibrogenesis, HO-1 may delay progression to hepatocellular carcinoma. CX3CR1þ macrophages in the intestinal lamina propria are considered to contribute to gut homeostasis through the immunomodulatory IL10 signaling [67]. Interaction of CX3CL1 with its receptor (CX3CR1) induced upregulation of HO-1 in the colon of mice. Thus, CX3CR1-deficient mice express HO-1 in the colonic mucosa at a much lower levels than the WT mice. Notably, pharmacologic inhibition HO-1 by ZnPP administration aggravated the dextran sulfate sodium-induced colitis, whereas the HO-1 inducer CoPP treatment ablated intestinal inflammation and fully protected CX3CR1 KO mice from azoxymethane and dextran sulfate sodium-induced colon carcinogenesis [67].

Gandini et al. reported that HO-1 overexpression reduced the tumor burden in two different murine models (syngeneic and xenograft) of breast cancer development and that positive expression of HO-1 was associated with longer overall survival of patients with breast cancer [68]. In contrast, Noh et al. showed a correlation between HO-1 expression and poor survival of breast cancer patients [69]. The association of aberrant HO-1 expression with tumor growth and resistance to therapy has been shown in other types of malignancies, such as human renal cell carcinoma [70], prostate [71] and pancreatic [10] cancers, lymphosarcoma [72], melanoma, and hepatoma [11]. Interestingly, autophagy induced by the activation of Src/STAT3/HO-1 was found to protect several subtypes of breast cancer cells from doxorubicin-induced cytotoxicity [73]. Furthermore, NRF2-dependent HO-1 induction rendered neuroblastoma cells resistant to bortezomib [74]. Such differential effects of HO-1 on cancer development and progression may depend on the subtype of breast cancer and tumor microenvironment. Subcellular localization of HO-1 is also important in differential functions in cancer. The nuclear accumulation of HO-1 has been postulated to have significant effects on the progression of various tumors [71,75]. Nuclear HO-1 has been reported to be associated with a higher histological grade [68,76].

Our demonstration of induction of HO-1 by 15d-PGJ_2_ in human breast cancer (MCF-7) cells is in agreement with several previous studies, implying the oncogenic potential of elevated HO-1. According to Fan et al. [77], the overexpression of HO-1 in endothelial cells caused a significant increase in angiogenesis. Similarly, the overexpression of HO-1 potentiated the invasiveness of pancreatic cancer by increasing tumor growth, angiogenesis, and metastasis [13]. In gastric cancer cells, elevated levels of HO-1 and p21 conferred resistance to apoptosis via MAPK-mediated NF-κB signaling [14]. Furthermore, the upregulation of HO-1 enhanced the VEGF synthesis in vascular smooth muscle cells [77,78]. The differential roles for HO-1 in angiogenesis, depending on the inflammatory stage, have also been reported [22]. As a potent mediator of vascular permeability, VEGF is an important angiogenic factor reported to induce recruitment and proliferation of endothelial cells [79]. In the present study, 15d-PGJ_2_ significantly stimulated vessel sprouting by inducing the elevated expression of VEGF. The upregulation of VEGF by 15d-PGJ_2_ could be mimicked by the induction of HO-1 expression. Moreover, inhibition of HO-1 abrogated the expression of VEGF and physiological angiogenic activity and significantly decreased the formation of tube-like structures in HUVECs on Matrigel and migrative behavior of MCF-7 cells stimulated with 15d-PGJ_2_. These results are in agreement with those of a previous study demonstrating the attenuation of the angiogenic activity by HO-1 inhibition [80].

Analysis of the VEGF promoter region reveals several potential binding sites for transcription factors AP-1, AP-2, SP-1, and HIF-1 [81]. Under normoxia, the basal and cytokine-enhanced VEGF expression is mediated mainly by the SP-1 protein, which interacts with four SP-1 binding sequences located in the proximity of the transcription initiation site [82]. In contrast, the most important transcription factor responsible for hypoxia-induced generation of VEGF is HIF-1 [83]. In this study, we demonstrated that HO-1 induced the expression of VEGF and physiological angiogenic activity. The expression of HO-1 is regulated through NRF2-ARE activation. We also found the involvement of NRF2 in 15d-PGJ_2_-induced upregulation of VEGF by use of dominant-negative vector and si-RNA knockdown of NRF2 gene. Furthermore, 15d-PGJ_2_ failed to induce angiogenesis via endothelial-cell sprouting in NRF2 deficient mice. These results suggest that NRF2 plays a crucial role in mediating 15d-PGJ_2_-induced expression of HO-1 and VEGF.

Although the nuclear translocation of NRF2 with subsequent binding to ARE/EpRE has been highly regarded as the critical prerequisite for the induction of ARE-dependent cytoprotective gene expression [84], the present study strongly suggests the possibility that NRF2 regulates angiogenesis and hence tumor progression under certain circumstances. In hypoxia, the induction of antioxidant enzymes activity through activation of NRF2 may provide survival advantage in cancerous cells or tissues [85]. The induction of HO-1 and VEGF mediated by NRF2 in the 15d-PGJ_2_-treated breast cancer cells appears to contribute to the manifestation of the angiogenic phenotype. In normal cells, acute activation of the NRF2 signaling protects against genotoxic insults, but its aberrant hyperactivation in (pre)malignant cells may support their survival, particularly in an environment in which there are high levels of ROS and/or electrophiles. In this context, NRF2 may act as a double-edged sword.

In this study, we also measured the level of 15d-PGJ_2_ in tumor tissues from breast cancer patients. Among the 18 tumor specimens analyzed, all exhibited higher levels of 15d-PGJ_2_ compared to normal surrounding tissues, suggesting that 15d-PGJ_2_ may play an important role in human breast carcinogenesis. In addition, immunohistochemical analysis revealed that the levels of COX-2, NRF2, and HO-1 were escalated according to the cancer stage. This observation suggests that the high level of 15d-PGJ_2_ formed as a consequence of COX-2 upregulation triggers NRF2 activation, leading to HO-1 induction, which may contribute to the angiogenesis through increased VEGF expression in the breast cancer. A major weakness of this study is the relatively small number of samples analyzed, which was due to the limited availability of suitable fresh-frozen breast cancer tissues. Nonetheless, there is the inverse correlation between the expression of COX-2/NRF2 and breast cancer survival rate in the Kaplan-Meier analysis of publicly available breast cancer data set of TCGA.

There has been increasing evidence for a causal relationship between inflammation and cancer [86]. Ansari et al. [87] have reported that a positive feedback loop between COX-2 and PGE_2_ is mediated by the EP2 receptor. As a final product of COX-2, 15d-PGJ_2_ may exert differential effects on inflammation and inflammation-associated carcinogenesis, depending on its intracellular concentrations. Our previous study has proposed the positive regulation of COX-2 by 15d-PGJ_2_ [88]. The malignant cells might employ these vicious loops for their own growth benefit and survival advantage. In addition, 15d-PGJ_2_ also increased the expression of HO-1 and VEGF as well as capillary formation and migration via the NRF2 signaling cascade in MCF-7 cells, which provides a mechanistic basis for the oncogenic potential of 15d-PGJ_2_. In several pathological conditions, prostaglandin levels in the micromolar range have been detected at the sites of acute inflammation [89]. We observed that the level of 15d-PGJ_2_ as well the expression of COX-2, NRF2, and HO-1 is increased in human breast cancer tissues. Therefore, the elevated levels of 15d-PGJ_2_ during inflammatory tissue damage are likely to provoke activation of NRF2, thereby upregulating HO-1 and hence VEGF expression. Taken together, these findings provide a novel mechanism underlying oncogenic function of 15d-PGJ_2_ that links COX-2-NRF2-HO-1-VEGF axis.

## Data Availability

All the data presented in this study are included in this article and its Supplementary Material file.

## Supplementary Materials

The following are available online at https://www.mdpi.com/2073-4409/10/3/526/s1. Table S1: Immunohistochemical analysis of COX-2, NRF2, and HO-1 in breast cancer tissues at different stages; Table S2: The ab initio calculation of 15d-PGJ_2_ and 9,10-dihydro-15d-PGJ_2_ to the thiol residues; Table S3: Functional categories enriched among differentially expressed genes in 15d-PGJ_2_ and 9,10-dihydro-15d-PGJ_2_-stimulated cells relative to control DMSO-stimulated cells. Figure S1: The expression of COX-2, NRF2, and HO-1 in human breast cancer tissues vs. surrounding normal tissues; Figure S2: The elevated levels of PGE_2_ in human breast cancer tissues.
