# Supplementary material for: 15-Deoxy-Δ12,14-prostaglandin J2 Upregulates VEGF Expression via NRF2 and Heme Oxygenase-1 in Human Breast Cancer Cells
Source: Cells. 2021 Mar 2;10(3):526. doi: 10.3390/cells10030526 (PMC8002112; doi:10.3390/cells10030526)
Supplement: Supplementary file 1 [file cells-10-00526-s001.pdf]

15-Deoxy-Δ[12, 14]-prostaglandin J_2 Upregulates VEGF Expression via NRF2 and Heme Oxygenase-1 in Human Breast Cancer Cells

Eun-Hee Kim [1,*], Su-Jung Kim [2], Hye-Kyung Na [3], Wonshik Han [4,5], Nam-Jung Kim [6], Young-Ger Suh [1], and Young-Joon Surh [2,4,7,*]

Supplementary materials:

Table S1. Immunohistochemical analysis of COX-2, Nrf2 and HO-1 in breast cancer tissues at different stages.

	No.	COX-2	Nrf2	HO-1
Normal	1	-	±	±
	2	-	±	±
	3	-	±	±
	4	-	±	±
	5	-	+	+
	6	-	±	±
	7	-	±	±
	8	-	±	±
	9	-	±	±
	10	-	±	±
	11	-	±	±
	12	-	±	±
	13	-	±	±
	14	-	±	±
	15	-	±	±

	stage	No.	COX-2	Nrf2	HO-1
Cancer	1	1	-	±	±
		2	-	±	±
	2	3	+	±	+
		4	-	±	+
		5	+	±	+
		6	+	±	+
		7	+	±	+
	3	8	+	+	+
		9	+	+	+ +
		10	+	+	+
		11	+	+ +	+ +
		12	+	+	+ +
		13	+	++	+ +
		14	+	+	+ +
		15	++	+	+ +
		16	++	++	+ +
	4	17	+ +	+	+ +
		18	+ +	+	+ +

Table S2. The *ab initio* calculation of 15d-PGJ2 and 9,10-dihydro-15d-PGJ2 to the thiol residues.

	Energy (Hartree)	Notes
$E^{CH_3S^-}$	-437.1149	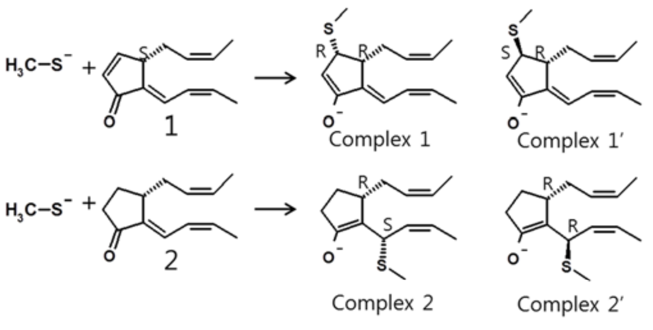
E^{1}	-576.4031	
E^{2}	-577.5868	
$E^{1}_{Complex}$	-1013.5179	
$E^{1'}_{Complex}$	-1013.5227	
$E^{2}_{Complex}$	-1014.6949	
$E^{2'}_{Complex}$	-1014.6837	
ΔE^{1}_{bind}	0.06 kcal/mol	(*X*= 1, 1', 2, 2') $\Delta E^{X}_{bind} = E^{X}_{Complex} - (E^{CH_3S^-} + E^{X})$
$\Delta E^{1'}_{bind}$	-2.95 kcal/mol	$\Delta E^{1'}_{bind} < \Delta E^{1}_{bind} < \Delta E^{2}_{bind} < \Delta E^{2'}_{bind}$
ΔE^{2}_{bind}	4.27 kcal/mol	
$\Delta E^{2'}_{bind}$	11.30 kcal/mol	

1. 1Hartree = 627.5095kcal/mol,
2. Calculation : Gaussian 98, Hartree-Fork, Restricted, 6-31G(d), optimization

Table S3. Functional categories enriched among differentially expressed genes in 15d-PGJ2 and 9,10-dihydro-15d-PGJ2-stimulated cells relative to control DMSO-stimulated cells. P, 15d-PGJ2; D, 9,10-dihydro-15d-PGJ2; C, control.

Category (Biological process)		Fold (P/C)	Fold (D/C)
Antioxidation and free radical removal /Detoxification /Stress response	heme oxygenase-1 (HO-1)	89.37 ± 0.11	5.26 ± 0.29
	heat shock 70kDa protein 6 (HSP70B') (HSPA6)	86.50 ± 0.28	-1.04 ± 0.10
	glutathione peroxidase 3 (GPX3)	8.91 ± 0.18	1.15 ± 0.15
	glutathione peroxidase 2 (GPX2)	4.76 ± 0.07	3.53 ± 0.07
	glutamate-cysteine ligase,	8.19 ± 0.07	2.79 ± 0.07
	glutamate-cysteine ligase,	3.21 ± 0.18	2.09 ± 0.20
	thioredoxin reductase 1	8.22 ± 0.11	2.89 ± 0.10
	glutathione reductase (GSR)	3.12 ± 0.08	1.49 ± 0.06
	FOS-like antigen 1 (FOSL1)	18.19 ± 0.12	1.11 ± 0.14
	cytochrome P450 (CYP4F11)	10.28 ± 0.15	2.66 ± 0.19
	growth arrest and DNA-damage-inducible, alpha	9.56 ± 0.10	1.21 ± 0.12
Cell structure and motility	GABA(A) receptor-associated protein like 1 (GABARAPL1)	29.57 ± 0.18	1.5 ± 0.37
	tubulin, alpha 1 (TUBA1)	15.28 ± 0.25	1.85 ± 0.28
	microtubule-associated protein 1B (MAP1B)	12.62 ± 0.10	1.07 ± 0.14
Cell proliferation and differentiation	FOS-like antigen 1 (FOSL1)	18.19 ± 0.12	1.11 ± 0.14
	v-maf musculoaponeurotic fibrosarcoma oncogene homolog	8.06 ± 0.11	1.82 ± 0.14
	epidermal growth factor (EGF)	4.72 ± 0.13	1.2 ± 0.11
	microtubule-associated protein (MAPRE3)	5.08 ± 0.11	1.2 ± 0.06
	microtubule-associated protein, (MAPRE2)	4.54 ± 0.11	1.19 ± 0.10

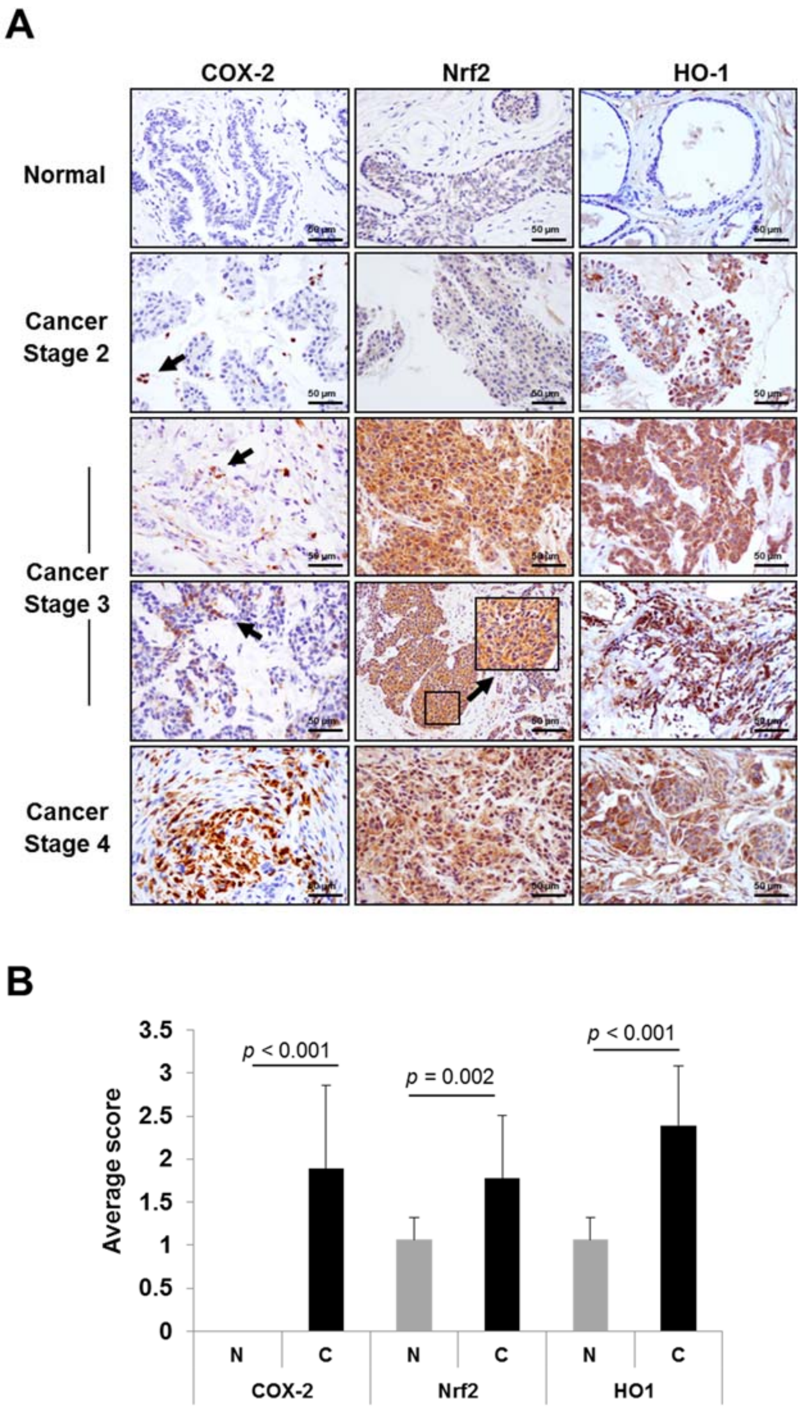

Figure S1. The expression of COX-2, Nrf2 and HO-1 in human breast cancer tissues *vs* surrounding normal tissues. (**A**) Paraffin-embedded human breast cancer at different stages or normal tissues were immunostained for COX-2, Nrf2 and HO-1, and counterstained with hematoxylin, as described in Materials and Methods. (**B**) The percentage of cells positive for COX-2, Nrf2 and HO-1 staining was scored in a blinded manner: 1, < 5% positive cells; 2, 5-20% positive; 3, 20-50% positive; 4, 50-80% positive; 5, > 80% positive. (n = 15 for the normal group (N) and n = 18 for the cancer group (C).

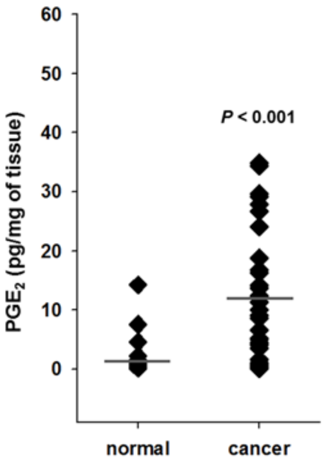

Figure S2. The elevated levels of PGE_2 in human breast cancer tissues. PGE_2 production in human breast cancer and normal tissues were measured as described in Materials and methods.
